# Impact of Increased Hemoglobin on Spontaneous Intracerebral Hemorrhage

**DOI:** 10.1007/s12028-021-01305-1

**Published:** 2021-07-27

**Authors:** Yuxuan Lu, Haiqiang Jin, Yuhua Zhao, Yuxian Li, Jun Xu, Jiayu Tian, Xiaoting Luan, Siwei Chen, Wei Sun, Shouzi Zhang, Shunliang Xu, Feiqi Zhu, Luzeng Chen, Dunzhu Mima, Yongan Sun, Cidan Zhuoga

**Affiliations:** 1 Department of Neurology, Peking University First Hospital, Beijing, China; 2 Department of Neurology, People’s Hospital of Tibet Autonomous Region, Lhasa, Tibet China; 3 Department of Cognitive Neurology, China National Clinical Research Center for Neurological Diseases, Beijing Tiantan Hospital, Capital Medical University, Beijing, China; 4 Department of Neurology, The First Hospital of Tsinghua University, Beijing, China; 5 Department of Psychiatry, Beijing Geriatric Hospital, Beijing, China; 6 Department of Neurology, the Second Hospital, Shandong University, Jinan, Shandong Province China; 7 Cognitive Impairment Ward of Neurology Department, the Third Affiliated Hospital of Shenzhen University Medical College, Shenzhen, China; 8 Department of Ultrasound, Peking University First Hospital, Beijing, China

**Keywords:** Cerebral hemorrhage, Hemoglobin, Risk factor, Hematoma volume, Hemorrhagic location

## Abstract

**Background:**

Studies of the impact of increased hemoglobin on spontaneous intracerebral hemorrhage (ICH) are limited. The present study aimed to explore the effect of increased hemoglobin on ICH.

**Methods:**

A retrospective single-center study using medical records from a database processed by univariate and multivariate analyses was performed in the People’s Hospital of Tibet Autonomous Region in Lhasa, Tibet, China.

**Results:**

The mean hemoglobin level in 211 patients with ICH was 165.03 ± 34.12 g/l, and a median hematoma volume was 18.5 ml. Eighty-eight (41.7%) patients had large hematomas (supratentorial hematoma ≥ 30 ml; infratentorial hematoma ≥ 10 ml). No differences in ICH risk factors between the groups with different hemoglobin levels were detected. Increased hemoglobin was independently associated with large hematomas [odds ratio (OR) 1.013, *P* = 0.023]. Increased hemoglobin was independently associated with ICH with subarachnoid hemorrhage (OR 1.014, *P* = 0.016), which was more pronounced in men (OR 1.027, *P* = 0.002). Increased hemoglobin was independently associated with basal ganglia hemorrhage and lobar hemorrhage in men (OR 0.986, *P* = 0.022; OR 1.013, *P* = 0.044, respectively) but not in women (*P* > 0.1).

**Conclusions:**

Increased hemoglobin was independently associated with large hemorrhage volume. Increased hemoglobin was independently associated with lobar hemorrhage in men and ICH with subarachnoid hemorrhage, which was more pronounced in men. Additional studies are needed to confirm our findings and explore potential mechanisms.

## Introduction

Spontaneous intracerebral hemorrhage (ICH) is a common neurological emergency, contributing the highest number of fatalities among all types of stroke. Overall annual incidence of ICH is 24.6 per 100,000, with a median case fatality of 40.4% at 1 month [1]. Previous studies reported that low hemoglobin levels are associated with poor outcomes in patients with ICH, and these patients are inclined to have larger hematomas [2–11]. However, studies focusing on associations of high hemoglobin levels with ICH, especially ICH volume and location, are limited. One of the main reasons for these limitations is that the data from patients with ICH with high hemoglobin levels are not accessible.

Patients living at high altitude, for example, in Tibet, have certain specific clinical characteristics because of long-term exposure to chronic hypoxia and adaptively high hemoglobin levels. Thus, we conducted the present study to explore the role of increased hemoglobin in patients with ICH.

## Methods

### Patients

The medical records of Tibetan patients (residing at altitudes between 3500 and 4000 m) with hemorrhagic stroke consecutively hospitalized between January 1, 2018, and December 31, 2018, were retrospectively recruited from the database of People’s Hospital of Tibet Autonomous Region. The data were reviewed and approved by the Ethics Committee for Human Research of the Peking University First Hospital. Oral consent was obtained from all patients at admission.

Complete data sets included demographics, clinical characteristics, medical history, baseline Glasgow coma scale (GCS) score, laboratory tests on admission, cranial computed tomography (CT) data on admission, interventions, and Modified Rankin Scale (mRS) at discharge were obtained for 242 patients.

The results of CT and computed tomography angiography (CTA), magnetic resonance angiography (MRA), or digital subtraction angiography, were used to exclude patients with primary subarachnoid hemorrhage (SAH), and patients with spontaneous ICH with intraventricular hemorrhage (IVH) and/or SAH were included. Finally, 31 patients were excluded: 22 patients with known or suspected secondary ICH (ischemic stroke with hemorrhagic transformation, vascular malformation, aneurysm, malignancy, and trauma), 3 patients with primary IVH, 5 patients with blurred CT images, and 1 patient without hemoglobin data. A total of 211 patients with primary ICH were included in the final analysis.

### Explanatory Variables

Hypertension was defined as systolic blood pressure ≥ 140 mm Hg and/or diastolic blood pressure ≥ 90 mm Hg at the time of clinical examination and/or current use of antihypertensive medications. Diabetes mellitus (DM) was defined as confirmed diagnosis of type 1 or type 2 DM. Diagnosis of coronary heart disease included myocardial infarction and angina and was confirmed based on the medical history. Smoking was defined as a self-reported current or past habit of smoking; alcohol use was defined as a self-reported current or past habit of drinking alcohol. Stroke history included previous ischemic stroke or hemorrhagic stroke. Antithrombotic agent history was defined as history of using antiplatelet or anticoagulant agents. The results of routine blood tests included hemoglobin, blood biochemistry, blood lipids, and coagulation function and were obtained on the first day of admission of the patients. External ventricular drain placement or surgical hematoma evacuation were also considered. Coma was divided into three degrees according to the GCS score (level 1: 13–15 points; level 2: 9–12 points; and level 3: 3–8 points) [12]. The hemoglobin level on admission was used to divide patients into two groups (the threshold value of the hemoglobin level was 165 g/l in men and 160 g/l in women) according to the World Health Organization diagnostic guidelines 2016 [14].

### Neuroimaging and Outcome Assessment

The results of cranial CT scanning on the first day of admission were processed by volumetric analysis using Scion Image software (Scion Corp, Frederick, MD), which is a public domain software modified based on National Institutes of Health (NIH) Image software (Scion Corporation, 2000–2001). Hemorrhages were traced on all head CT slices, and the volumes were calculated by multiplying by thickness of the slices [15]. Primary SAH was defined as SAH (diagnosed based on head CT) that resulted from a ruptured aneurysm or arteriovenous malformation (confirmed by CTA, MRA, or digital subtraction angiography), and the patients with primary SAH were excluded [16]. Lobar ICH with hematoma irrupting into the subarachnoid space was included. The location of ICH and the presence or absence of IVH or SAH were assessed based on the results of initial head CT by a neurologist who was blinded to the goals of the present study according to anatomic location predominantly engaged by ICH [17]. Simultaneous multiple ICH (SMICH) were defined as two or more acute discrete, noncontiguous intraparenchymal hematomas detected by initial diagnostic CT [18]. If a single contiguous hematoma was too large and hard to locate and involved several locations or if ICH was defined as SMICH, all affected locations were included. Large hematoma was defined as hemorrhage with a volume ≥ 30 ml for supratentorial hemorrhage or ≥ 10 ml for infratentorial hemorrhage [19, 20]. Functional outcome at discharge was assessed by mRS. GCS and mRS were scored by attending physicians, whom have been certified by systematic training, with excellent accuracy. The discharge outcome scores were dichotomized into favorable (mRS 0–3) and poor functional outcomes (mRS 4–6).

### Statistical Analysis

Categorical variables are presented as percentages. Numerical variables are presented as the mean ± standard deviation or median [interquartile range (IQR)]. The *χ*^2^ test was used to compare categorical variables, and Student’s *t*-test or Wilcoxon rank-sum test were used to compare numerical variables based on whether distributions of the variables were normal or not normal, respectively. Variables with a *P* value < 0.1 in univariate analysis were included into subsequent multivariate analysis for large hematoma and hemorrhagic locations. Variables with a *P* value < 0.05 in multivariate analysis were considered statistically significant. Sensitivity analysis of associations between the level of hemoglobin and large hematoma included the locations of lobar hemorrhage alone and of the complex of the basal ganglia, lobe, and thalamus used in the multivariate analysis model for large hemorrhage as described above. A scatterplot was generated, and the correlation coefficients were determined to assess the associations between hemoglobin level and hematoma volume. Statistical analyses were performed using International Business Machines Corporation (IBM) Statistical Product and Service Solutions (SPSS) 21.0 software (IBM, Armonk, NY).

## Results

### Characteristics of the Cohort of Patients with ICH

The study cohort included 211 patients with a mean age of 58.34 ± 12.08 years, and 142 (67.3%) patients were men. The cohort included 151 (71.6%) patients with hypertension, 3 (1.4%) patients with DM, 13 (6.2%) patients with coronary heart disease, and 17 (8.1%) patients with stroke history; the median GCS score was 12. Six patients (2.8%) reported the use of antiplatelet agents, and none of the patients reported the use of anticoagulant agents prior to admission. Regarding location, 82 (38.9%) patients had hemorrhage of the basal ganglia, 66 (31.3%) patients had lobar hemorrhage, 54 (25.6%) patients had hemorrhage of the thalamus, 16 (7.6%) patients had hemorrhage of the brainstem, and 14 (6.6%) patients had hemorrhage of the cerebellum. In the whole cohort, 18 (8.5%) patients had hemorrhage with mixed locations, of whom 7 (3.3%) patients had SMICH. These 18 patients included 3 (1.4%) patients with hemorrhage involving three locations and 15 (7.1%) patients with hemorrhage involving two locations. Hemorrhage with mixed locations involving the thalamus and basal ganglia was the major type and occurred in ten patients (4.7%). A total of 106 (50.2%) patients had ICH with IVH, and 35 (16.6%) patients had ICH with SAH. Among these 35 patients, 18 patients (51.4%) had cortical SAH and 17 patients (48.6%) had basal cistern SAH. Furthermore, the median hematoma volume was 18.50 ml (IQR 7.94–43.70 ml); 88 (41.7%) patients had large hematoma; 45 (21.3%) patients underwent surgery, and 89 (42.2%) patients had mRS of 4–6 at discharge (Table 1).

**Table 1:** Characteristics of 211 patients with ICH in Tibet

Variables	Study participants (*n* = 211)
Age, mean ± SD (year)	58.34 ± 12.08
Male sex [*n* (%)]	142 (67.3)
BMI, median (IQR) (kg/m^2^)	23.39 (22.26–24.66)
Smoking history [*n* (%)]	38 (18.0)
Alcohol history [*n* (%)]	27 (12.8)
Hypertension [*n* (%)]	151 (71.6)
Diabetes mellitus [*n* (%)]	3 (1.4)
Coronary heart disease [*n* (%)]	13 (6.2)
Stroke history [*n* (%)]	17 (8.1)
Antithrombotic agent history [*n* (%)]	6 (2.8)
The degree of coma (GCS score) [*n* (%)]	
Level 1 (13–15 points)	96 (45.5)
Level 2 (9–12 points)	41 (19.4)
Level 3 (3–8 points)	74 (35.1)
Supratentorial hemorrhage [*n* (%)]	
Basal ganglia	82 (38.9)
Lobar	66 (31.3)
Thalamus	54 (25.6)
Infratentorial hemorrhage [*n* (%)]	
Brainstem	16 (7.6)
Cerebellum	14 (6.6)
IVH [*n* (%)]	106 (50.2)
SAH [*n* (%)]	35 (16.6)
Large hematoma [*n* (%)]	88 (41.7)
Hematoma volume, median (IQR) (ml)	18.50 (7.94–43.70)
Hematoma volume at various hemorrhagic locations	
Basal ganglia, median (IQR) (ml)	24.38 (12.86–50.40)
Lobar, median (IQR) (ml)	22.33 (9.64–47.63)
Thalamus, median (IQR) (ml)	17.72 (7.34–43.53)
Brainstem, median (IQR) (ml)	7.05 (2.86–15.34)
Cerebellum, median (IQR) (ml)	11.25 (5.91–25.22)
Laboratory examination	
TG, median (IQR) (mmol/l)	0.95 (0.73–1.36)
HDL, mean ± SD (mmol/l)	1.21 ± 0.34
LDL, mean ± SD (mmol/l)	2.43 ± 0.91
TC, mean ± SD (mmol/l)	4.07 ± 1.11
Hemoglobin, mean ± SD (g/l)	165.03 ± 34.12
Platelet count, mean ± SD (10^9^/l)	188.31 ± 71.49
Surgical operation [*n* (%)]	45 (21.3)
mRS 4–6 [*n* (%)]	89 (42.2)

*BMI* Body mass index, *GCS* Glasgow Coma Scale, *HDL* high-density lipoprotein cholesterol, *ICH* intracerebral hemorrhage, *IQR* interquartile range, *IVH* intraventricular hemorrhage, *LDL* low-density lipoprotein cholesterol, *mRS* Modified Rankin Scale, *SAH* subarachnoid hemorrhage, *SD* standard deviation, *TC* total cholesterol, *TG* triglycerides

The group with normal/low hemoglobin level included 114 patients, and 97 patients were included in the group with elevated hemoglobin level. No differences in ICH risk factors were detected between the two groups (*P* > 0.05) except sex (*P* = 0.001). The elevated hemoglobin level group tended to have large hematoma (*P* = 0.007) (Table 2).

**Table 2:** Characteristics of various ICH subgroups divided according to hemoglobin level

Variable	Normal/low hemoglobin level group (*n* = 114)	Elevated hemoglobin level group (*n* = 97)^a^	*P* value
Age, mean ± SD (year)	59.64 ± 12.59	56.81 ± 11.34	0.091^b^
Male sex [*n* (%)]	65 (57.0)	77 (79.4)	0.001^c^
BMI, median (IQR) (kg/m^2^)	23.20 (21.72–24.90)	23.61 (22.53–24.53)	0.242^d^
Smoking history [*n* (%)]	22 (19.3)	16 (16.5)	0.597^c^
Alcohol history [*n* (%)]	13 (11.4)	14 (14.4)	0.511^c^
Hypertension [*n* (%)]	81(71.1)	70 (72.2)	0.858^c^
Diabetes mellitus [*n* (%)]	1 (0.9)	2 (2.1)	0.888^c^
Coronary heart disease [*n* (%)]	10 (8.8)	3 (3.1)	0.155^c^
Stroke history [*n* (%)]	11 (9.6)	6 (6.2)	0.357^c^
Antithrombotic agent history [*n* (%)]	4 (3.5)	2 (2.1)	0.830^c^
The degree of coma (GCS score) [*n* (%)]			0.161c^b^
Level 1 (13–15 points)	52 (45.6)	44 (45.4)	
Level 2 (9–12 points)	27 (23.7)	14 (14.4)	
Level 3 (3–8 points)	35 (30.7)	39 (40.2)	
Supratentorial hemorrhage [*n* (%)]			
Basal ganglia	46 (40.4)	36 (37.1)	0.631^c^
Lobar	33 (28.9)	33 (34.0)	0.428^c^
Thalamus	32 (28.1)	22 (22.7)	0.371^c^
Infratentorial hemorrhage [*n* (%)]			
Brainstem	7 (6.1)	9 (9.3)	0.391^c^
Cerebellum	8 (7.0)	6 (6.2)	0.809^c^
IVH [*n* (%)]	53 (46.5)	53 (54.6)	0.238^c^
SAH [*n* (%)]	13 (11.4)	22 (22.7)	0.028^c^
Large hematoma [*n* (%)]	38 (33.3)	50 (51.5)	0.007^c^
Hematoma volume, median (IQR) (ml)	17.31 (7.25–39.07)	21.66 (10.04–52.49)	0.096^d^
Laboratory examination			
TG, median (IQR) (mmol/l)	0.96 (0.75–1.38)	0.95 (0.72–1.35)	0.889^d^
HDL, mean ± SD (mmol/l)	1.22 ± 0.34	1.20 ± 0.35	0.729^b^
LDL, mean ± SD (mmol/l)	2.24 ± 0.84	2.55 ± 0.99	0.094^b^
TC, mean ± SD (mmol/l)	3.99 ± 1.03	4.16 ± 1.19	0.284^b^
Hemoglobin, mean ± SD (g/l)	140.67 ± 17.26	193.66 ± 25.75	< 0.001^b^
Platelet count, mean ± SD (10^9^/l)	195.21 ± 78.72	180.20 ± 61.35	0.129^b^
mRS 4–6 [*n* (%)]	48 (42.1)	41 (42.3)	0.981^c^

*BMI* body mass index, *GCS* Glasgow Coma Scale, *HDL* high-density lipoprotein cholesterol, *ICH* intracerebral hemorrhage, *IQR* interquartile range, *IVH* intraventricular hemorrhage, *LDL* low-density lipoprotein cholesterol, *mRS* Modified Rankin scale, *SAH* subarachnoid hemorrhage, *SD* standard deviation, *TC* total cholesterol, *TG* triglycerides

^a^Elevated hemoglobin was defined as hemoglobin ≥ 165 g/l in men and ≥ 160 g/l in women

^b^Student’s *t*-test

^c^*χ*2 test

^d^Mann–Whitney *U*-test

### Factors Associated with the Volume and Location of Hematoma

**Fig. 1:**
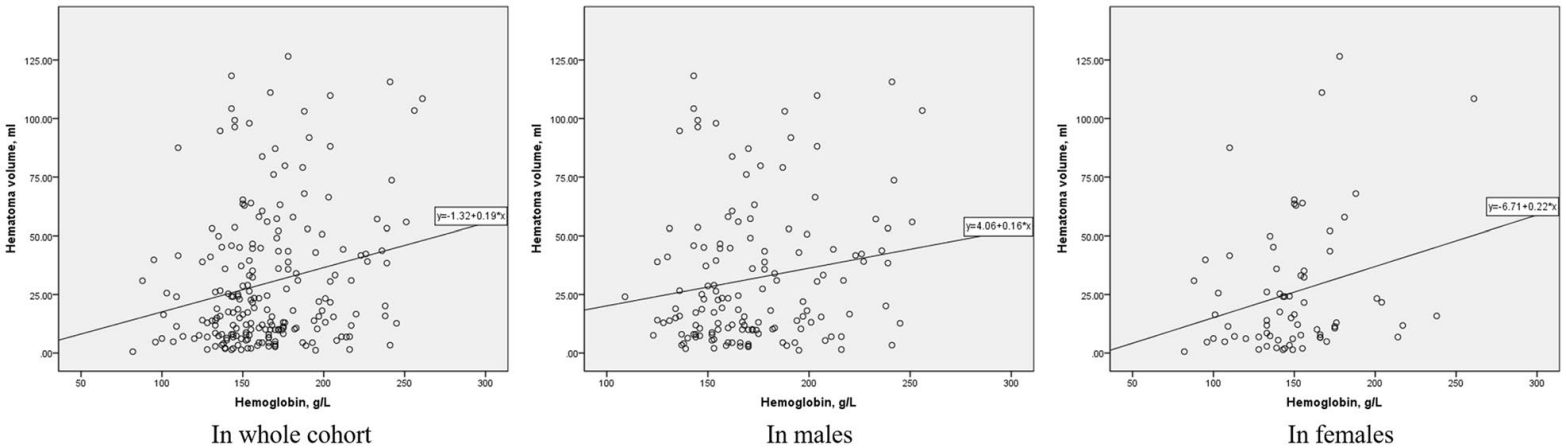
Scatterplot of hemoglobin levels and hematoma volumes

The results of univariate analysis indicated that smoking history (13.0% vs. 25.0%, *P* = 0.025), alcohol history (8.9% vs. 18.2%, *P* = 0.048), hypertension (78.9.0% vs. 61.4%, *P* = 0.005), IVH (32.5% vs. 75%, *P* < 0.001), SAH (8.1% vs. 28.4%, *P* < 0.001), triglycerides [1.00 (IQR 0.81–1.43) mmol/l vs. 0.87 (IQR 0.64–1.25) mmol/l, *P* = 0.011], high-density lipoprotein cholesterol (1.16 ± 0.34 mmol/l vs. 1.28 ± 0.35 mmol/l, *P* = 0.012), hemoglobin (157.95 ± 29.86 g/l vs. 174.92 ± 37.29 g/l, *P* = 0.001), and platelet count (201.19 ± 77.03 × 10^9^/l vs. 170.31 ± 58.77 × 10^9^/l, *P* = 0.001) were significantly different between the group with mild to moderate hematoma and the group with large hematoma. The results of multivariate analysis indicated that IVH (*P* < 0.001, OR 6.796), high-density lipoprotein cholesterol (*P* = 0.019, OR 3.839), hemoglobin (*P* = 0.023, OR 1.013), and platelet count (*P* = 0.001, OR 0.990) were independent predictive factors for large hematoma. The results of sensitivity analysis indicated that hemoglobin level remained an independent predictive factor (in the model including lobar hemorrhage alone: *P* = 0.038, OR 1.012; in the model including complex locations: *P* = 0.039, OR 1.012) (Tables 3, 4; Fig. 1).

**Table 3:** Identification of risk factors for large hematoma by univariate analysis

Variable	Mild to moderate hematoma (*n* = 123)	Large hematoma (*n* = 88)	*P* value
Age, mean ± SD (year)	56.57 ± 11.61	56.49 ± 12.55	0.059^a^
Male sex [*n* (%)]	79 (64.2)	63 (71.6)	0.261^b^
BMI, median (IQR) (kg/m^2^)	23.30 (21.95–24.51)	23.77 (22.44–24.96)	0.226^c^
Smoking history [*n* (%)]	16 (13.0)	22 (25.0)	0.025^b^
Alcohol history [*n* (%)]	11 (8.9)	16 (18.2)	0.048^b^
Hypertension [*n* (%)]	97 (78.9)	54 (61.4)	0.005^b^
Diabetes mellitus [*n* (%)]	2 (1.6)	1 (1.1)	1.000^b^
Coronary heart disease [*n* (%)]	6 (4.9)	7 (8.0)	0.359^b^
Stroke history [*n* (%)]	8 (6.5)	9 (10.2)	0.327^b^
Antithrombotic agent history [*n* (%)]	4 (3.3)	2 (2.3)	0.998^b^
Supratentorial hemorrhage [*n* (%)]			
Basal ganglia	45 (36.6)	37 (42.0)	0.422^b^
Lobar	37 (30.1)	29 (33.0)	0.657^b^
Thalamus	32 (26.0)	22 (25.0)	0.868^b^
Infratentorial hemorrhage [*n* (%)]			
Brainstem	9 (7.3)	7 (8.0)	0.863^b^
Cerebellum	7 (5.7)	7 (8.0)	0.515^b^
IVH [*n* (%)]	40 (32.5)	66 (75)	< 0.001^b^
SAH [*n* (%)]	10 (8.1)	25 (28.4)	< 0.001^b^
Laboratory examination			
TG, median (IQR) (mmol/l)	1.00 (0.81–1.43)	0.87 (0.64–1.25)	0.011^c^
HDL, mean ± SD (mmol/l)	1.16 ± 0.34	1.28 ± 0.35	0.012^a^
LDL, mean ± SD (mmol/l)	2.50 ± 0.94	2.34 ± 0.87	0.215^a^
TC, mean ± SD (mmol/l)	4.08 ± 1.14	4.05 ± 1.07	0.811^a^
Hemoglobin, mean ± SD (g/l)	157.95 ± 29.86	174.92 ± 37.29	0.001^a^
Platelet count, mean ± SD (10^9^/l)	201.19 ± 77.03	170.31 ± 58.77	0.001^a^

*BMI* body mass index, *HDL* high-density lipoprotein cholesterol, *IQR* interquartile range, *IVH* intraventricular hemorrhage, *LDL* low-density lipoprotein cholesterol, *SAH* subarachnoid hemorrhage, *SD* standard deviation, *TC* total cholesterol, *TG* triglycerides

^a^Student’s *t*-test

^b^*χ*^2^ test

^c^Mann–Whitney *U*-test

**Table 4:** Identification of risk factors for large hematoma by multivariate analysis

Variables	*P* value	OR (95% CI)
Age (year)	> 0.05	0.981 (0.953–1.010)
Smoking history	> 0.05	1.262 (0.464–3.432)
Alcohol history	> 0.05	2.655 (0.900–7.833)
Hypertension	> 0.05	0.528 (0.247–1.125)
IVH	< 0.001	6.796 (3.197–14.446)
SAH	> 0.05	1.534 (0.556–4.237)
TG (mmol/l)	> 0.05	0.735 (0.436–1.240)
HDL (mmol/l)	0.019	3.839 (1.243–11.860)
Hemoglobin (g/l)	0.023	1.013 (1.002–1.024)
Platelet count (10^9^/l)	0.001	0.990 (0.984–0.996)

*CI* confidence interval, *HDL* high-density lipoprotein cholesterol, *IVH* intraventricular hemorrhage, *OR* odds ratio, *SAH* subarachnoid hemorrhage, *TG* triglycerides

In the male cohort, hemoglobin levels were significantly associated with basal ganglia hemorrhage (*P* = 0.022, OR 0.986) and with lobar hemorrhage (*P* = 0.044, OR 1.013). This association was not detected in the whole cohort or the female cohort. In the whole cohort, hemoglobin level was significantly different between the groups with hemorrhage with or without SAH (*P* = 0.016, adjusted OR 1.014), and basal ganglia hemorrhage and lobar hemorrhage were significantly different between the groups with hemorrhage with or without SAH according to univariate analysis (*P* < 0.001, OR 0.337; *P* = 0.005, OR 2.824, respectively); similar differences were not detected by multivariate analysis. Thalamus hemorrhage did not presented differences between the groups with hemorrhage with or without SAH. In the male cohort, the differences in hemoglobin level between the groups with hemorrhage with or without SAH were more pronounced (*P* = 0.002, adjusted OR 1.027), and the groups with basal ganglia hemorrhage and lobar hemorrhage were significantly different between the groups with hemorrhage with or without SAH according to the results of univariate analysis (*P* = 0.013, OR 0.282; *P* = 0.012, OR 3.005, respectively). Similar differences were not detected by multivariate analysis. Thalamus hemorrhage did not presented differences between the groups with hemorrhage with or without SAH. Similar differences in hemoglobin were not detected in the female cohort (Table 5).

**Table 5:** Univariate and multivariate analysis of associations of hemoglobin with hemorrhage locations

Sex	Basal ganglia group	Nonbasal ganglia group	*P* value	Adjusted *P* value	Adjusted OR (95% CI)
Male	165.67 ± 28.98	178.23 ± 32.62	0.019	0.022	0.986 (0.975–0.998)
Female	154.36 ± 28.66	146.17 ± 35.50	> 0.05	None^a^	None^a^
Total	162.63 ± 29.15	166.55 ± 36.96	> 0.05	None^a^	None^a^

Sex	Lobar group	Nonlobar group	*P* value	Adjusted *P* value	Adjusted OR (95% CI)
Male	181.43 ± 32.42	169.10 ± 30.69	0.031	0.044	1.013 (1.000–1.026)
Female	150.45 ± 43.00	148.00 ± 28.49	> 0.05	None^a^	None^a^
Total	171.11 ± 38.85	162.26 ± 31.50	> 0.05	None^a^	None^a^

Sex	Thalamus group	Nonthalamus group	*P* value	Adjusted *P* value	Adjusted OR (95% CI)
Male	172.09 ± 31.81	173.16 ± 31.74	> 0.05	None^a^	None^a^
Female	143.14 ± 30.60	151.43 ± 34.75	> 0.05	None^a^	None^a^
Total	160.30 ± 34.19	166.66 ± 34.06	> 0.05	None^a^	None^a^

Sex	IVH group	Non-IVH group	*P* value	Adjusted *P* value	Adjusted OR (95% CI)
Male	179.60 ± 34.97	166.78 ± 27.05	0.017	> 0.05	1.010 (0.996–1.024)
Female	148.68 ± 30.98	148.90 ± 36.85	> 0.05	None^a^	None^a^
Total	168.52 ± 36.61	161.50 ± 31.18	> 0.05	None^a^	None^a^

Sex	SAH group	Non-SAH group	*P* value	Adjusted *P* value	Adjusted OR (95% CI)
Male	198.36 ± 36.44	167.49 ± 27.81	< 0.001	0.002	1.027 (1.010–1.045)
Female	145.60 ± 48.29	149.32 ± 30.83	> 0.05	None^a^	None^a^
Total	183.29 ± 46.25	161.40 ± 30.03	0.010	0.016	1.014 (1.003–1.026)

Hemoglobin is presented as the mean ± standard deviation (g/L)

*CI* confidence interval, *IVH* intraventricular hemorrhage, *OR* odds ratio, *SAH* subarachnoid hemorrhage

^a^The variable with a *P* value > 0.1 in univariate analysis was not included in multivariate analysis, and the results are presented as “None”

## Discussion

### The Characteristics of ICH Cohorts

Previous studies in China reported that the mean age of patients with ICH was 57–62 years, and men accounted for 61.3–67.8% of patients with ICH [21–24]. The present study had similar characteristics for age and sex. The proportion of patients with GCS scores from 3 to 8 was reported to be 24.3% [23]. Compared with this study, the onset symptoms were more severe in the present study, and the proportion of patients with GCS scores from 3 to 8 was 35.1%. Previous studies reported that the median hematoma volume was 12.6 ml in a Chinese ICH population, and that the IVH rate of ICH was 29.6–45% at low altitude [23, 25–27]. In the present study, the hematoma volume was larger with a slightly higher rate of IVH. The basal ganglia was the most common hemorrhagic location reported previously, with a proportion of 34.2–57.9% [22, 23], and the proportion of basal ganglia hemorrhage in the present study was in agreement with that reported in previous studies. The discharge outcome of patients with ICH in the present study was similar to that in previous studies in the plains region, which reported that the proportion of patients with of ICH with mRS from 4 to 6 was 42.2–67.3% at discharge [4, 28]. No other differences in ICH risk factors were detected between the groups with different hemoglobin levels except sex. Previous studies reported a rate of stroke history varying from 2.0 to 9.0% in patients with acute stroke, and a few studies reported the rate of antithrombotic medication use [29–32]. The mismatch in the stroke history rate and antithrombotic medicine use in the present study may be caused by insufficient stroke rehabilitation system and inadequate realization of secondary prevention of stroke [29–31, 33].

### The Role of Hemoglobin in Hematoma Volume and Hemorrhagic Location

The results of the present study indicated that increased hemoglobin was associated with hematoma volume and was an independent risk factor for large hematoma. The presence of anemia was reported to result in a 39% increase in ICH volume [10]. A previous study suggested that a low hemoglobin level mediates a large ICH volume via altered mechanisms of hemostasis associated with a decrease in the red blood cell count mediated by an impairment of the platelet–platelet interactions and a decrease in the platelet–endothelium interactions, and impairment of the compensatory response of the cerebral vessels due to changes in hemodynamics also played a role [10]. Whether these mechanisms play important roles in associations of increased hemoglobin with ICH requires additional investigations.

Generally, hematoma volume is influenced by ICH location and is usually larger in lobar ICH than in deep ICH, which reflects different biological pathways. A previous study reported that ICH volume is associated with ICH location, intensity of anticoagulation, coronary artery disease, sex and age, and antiplatelet therapy [19]. Blood pressure at admission was associated with ICH volume, and early lowering of blood pressure may avoid hematoma enlargement [34, 35]. Regarding the effect of blood pressure at admission on hematoma volume in a specific hemorrhagic location, a study demonstrated by univariate analysis that blood pressure at admission influenced deep ICH volume, and a similar effect on lobar ICH volume was not detected [19]. This result indicates that deep ICH volume may be more susceptible to the effect of blood pressure at admission than lobar ICH volume. Blood pressure could have had important effects on the ICH volume in the deep and lobe in the present study, which differed from the expected results, in addition to potential influence of hemoglobin level. A previous study reported that patients with ICH in Tibet were inclined to have higher blood pressure at admission compared with that of other patients [30]. This parameter may influence deep ICH volume more than lobar ICH volume. The volume in mixed locations tended to be related to the volume in the case of deep ICH and may also contribute to this result. Thus, we suggest that the hemoglobin level is associated with large hematoma. Considering limited sample size, studies with larger sample sizes are needed to further explore the associations between hemoglobin level and ICH volume in specific locations.

Patients with lobar hemorrhage had significantly higher levels of hemoglobin. It is unclear why high hemoglobin levels are associated with higher predisposition in lobar ICH. A previous study reported that low arterial oxygen saturation and chronic obstructive pulmonary disease are associated with white matter lesions (WMLs), which were caused by high hemoglobin levels, leading to changes in cerebral blood flow mediated by hemodynamic changes [36]. It was unknown whether cerebral small vessel disease (CSVD), such as cerebral amyloid angiopathy and WMLs, was involved in this process, which may be mediated by hemoglobin levels or chronic hypoxia. Therefore, associations between hemoglobin, ICH, and CSVD remain in need of exploration. Increased hemoglobin levels were associated with differences between patients with ICH with or without IVH in the male cohort. This difference was eliminated by adjustment for basal ganglia hemorrhage and hematoma volume, which indicated that IVH was caused by large hematoma irruption in the lateral ventricle occurring in the basal ganglia.

Increased hemoglobin level was an independent risk factor for ICH with SAH, and this difference was more pronounced in the male cohort. Similar sex-dependent differences were also detected in associations of hemoglobin with ICH outcomes [6], and we suggest that these sex differences should be carefully assessed in future studies on ICH and hemoglobin. Associations between ICH with SAH and high hemoglobin levels have been rarely studied. In the present study, considerable part of SAH was resulted from redistribution of blood from IVH due to the deep ICH with large hematoma. We have excluded ICH caused by large arterial aneurysm; however, microaneurysms are difficult to detect by CTA or MRA and may also play a role in this process, even though the risk of rupture of these microaneurysms is extremely low. Previous studies reported that obstructive sleep apnea is associated with SAH due to enlarged arterial aneurysm and increased rupture rate, which is mediated by changes in the vessel wall and hemodynamics due to chronic hypoxia, systemic inflammation, and disturbance of autonomic nerves [37, 38]. Large hematomas irrupting in the subarachnoid space occurring in the cerebral lobe should also be considered.

Whether other risk factors, such as control of hypertension, may influence associations between hemoglobin level and ICH characteristics in the present study remains to be determined. Previous studies reported that diagnosis of hypertension and control of hypertension were associated with altitude, which presented the similar characteristics as hemoglobin to altitude [39, 40]. However, hypertension-related ICH tended to occur in the basal ganglia and thalamus, and these ICH locations were not significantly different between the groups with various ICH volumes. Hypertension history also did not significantly differ between the groups with various hemoglobin levels. Thus, there is a lack of evidence to prove that hypertension may interfere with associations between hemoglobin levels and ICH characteristics in the present study. Race was reported to be associated with ICH outcome and was not associated with significant differences in ICH volume [29]. However, this study included only Tibetan patients with ICH transported to the hospitals in low-altitude regions [29].

The present study has certain limitations. First, the present study is a single-center study with a limited sample size. However, considering the difficulty of recruitment of patients with ICH with increased hemoglobin, we think that the present study provides insight into relevant issues, and associations between hemoglobin and ICH need to be confirmed using a larger sample size. Second, the present study was conducted in a high-altitude region, and associations between high hemoglobin and ICH characteristics should be confirmed in a low-altitude region for generalization. Finally, the lack of CSVD evaluation, such as WMLs and cerebral microbleeds, resulted in uncertain associations between hemoglobin, ICH characteristics, and CSVD. Additional studies are needed to explore this issue.

## Conclusions

In conclusion, increased hemoglobin was independently associated with large hemorrhage volume. Moreover, increased hemoglobin was associated with lobar hemorrhage in men, and it was associated with and ICH with SAH that was more pronounced in male patients. Additional studies are needed to verify our findings and explore potential mechanisms of these effects.
